# Coelenterazine-Type Bioluminescence-Induced Optical Probes for Sensing and Controlling Biological Processes

**DOI:** 10.3390/ijms24065074

**Published:** 2023-03-07

**Authors:** Tianyu Jiang, Jingwen Song, Youming Zhang

**Affiliations:** 1 Helmholtz International Lab for Anti-Infectives, Shandong University–Helmholtz Institute of Biotechnology, State Key Laboratory of Microbial Technology, Shandong University, Qingdao 266237, China; 2 Shenzhen Research Institute of Shandong University, Shenzhen 518000, China; 3 School of Life Sciences, Shandong University, Qingdao 266237, China; 4 Chinese Academy of Sciences (CAS) Key Laboratory of Quantitative Engineering Biology, Shenzhen Institute of Synthetic Biology, Shenzhen Institute of Advanced Technology, Chinese Academy of Sciences, Shenzhen 518055, China

**Keywords:** bioluminescence, probes, coelenterazine, biosensors, optogenetics, cellular activity, signaling pathway, drug screening, gene therapy

## Abstract

Bioluminescence-based probes have long been used to quantify and visualize biological processes in vitro and in vivo. Over the past years, we have witnessed the trend of bioluminescence-driven optogenetic systems. Typically, bioluminescence emitted from coelenterazine-type luciferin–luciferase reactions activate light-sensitive proteins, which induce downstream events. The development of coelenterazine-type bioluminescence-induced photosensory domain-based probes has been applied in the imaging, sensing, and control of cellular activities, signaling pathways, and synthetic genetic circuits in vitro and in vivo. This strategy can not only shed light on the mechanisms of diseases, but also promote interrelated therapy development. Here, this review provides an overview of these optical probes for sensing and controlling biological processes, highlights their applications and optimizations, and discusses the possible future directions.

## 1. Introduction

Based on emissions from the luciferase oxidation of luciferin, bioluminescence-derived noninvasive tools have been used to image, sense, and investigate biological processes in vivo [1]. In nature, a diverse range of luciferases have the ability of oxidizing individual luciferins, causing them to reach light-emitting electronic states, which leads to photon generation [2]. Currently, nine different luciferase–luciferin systems with distinct wavelengths, co-factor dependencies, quantum yields, and other properties are used as bioluminescent toolkits in the fields of biology and medicine [2,3].

Bioluminescence reporters appear to be the first application of a tool derived from bioluminescence since the discovery of the first firefly luciferase–D-luciferin pair [4,5,6]. Luciferases have been routinely used in cell tracking and gene expression for a long time [1,2]. A biosensor is an analytical device composed of a biological sensing element and a transducer. The former is used to identify the target substance, while the latter is responsible for converting the interaction between the sensing element and the target molecule into different measurable signals [7,8]. With the in-depth elucidation of bioluminescence, a series of novel biosensors induced by bioluminescence have been designed and reported, and have gradually become a powerful tool for understanding complex biospecific interactions [1]. More recently, scientists have been focusing on the development of new synthetic luciferins, engineered luciferases, probes, and sensors, which not only sheds light on biological processes, but also expands the scope of bioluminescence techniques [1,3,9,10,11,12,13]. The bioluminescence developments of the past few years include a biological light source that allows optogenetic platforms to control signaling pathways and cellular activities. Optogenetics takes advantage of numerous light-activated proteins originating from nature to probe and control biological processes with external light illumination [14,15,16]. Luminopsin seems to be the first coelenterazine-type bioluminescence-induced optical probe [17]. This strategy provides an intrinsic light source and alternative optical modulation in optogenetics and has been successfully applied in neuroscience. However, this strategy not only is popular in neuroscience, but has also gradually come to play a role in other biological areas. We have observed the use of these tools and platforms in investigating biological processes, including sensing protein–protein interactions [18,19] and cell–cell communications [20], controlling signaling pathways [21], and even enabling synthetic biological activities [22,23]. The coupling of bioluminescent systems to light-inducible proteins has been considered to be a novel strategy by which to refactor gene circuits, with this approach sensing endogenous light to drive biological functions [9,24]. We think it is a promising trend to design and expand bioluminescence-induced optical tools for sensing and controlling involved in life science.

Here, we highlight coelenterazine-type bioluminescence-induced photosensory domain-based optical probes for sensing and controlling biological processes, and their applications in physiology and pathology, and potential therapy reported in the past 10 years. We also discuss future directions and the possible application of such tools in drug discovery and therapy development.

### 1.1. Bioluminescence and Bioluminescence Technology

Bioluminescence, as a form of chemiluminescence, is a natural phenomenon that emits cold light resulting from the luciferase-catalyzed reactions or photoproteins in biological systems [2]. Dozens of luciferase–luciferin pairs share similar mechanisms and emit bioluminescence but differ in their emission wavelengths and other optical properties; hence, they are suitable for various applications.

Coelenterazine (CTZ), as a luciferin, is utilized by many luciferases from marine lifeforms such as Renilla, Gaussia, and Oplophorus to yield blue-to-cyan light without any co-factor [4]. It is also the substrate for calcium-binding photoproteins, which results in the generation of a natural calcium indicator. The CTZ–luciferase system is the most common bioluminescence system to be merged with optogenetics according to recent developments discussed in detail in Section 2. It is likely that the blue emission from the CTZ–luciferase system is fitting for the absorption spectra of some photosensitive domains such as the light-oxygen-voltage (LOV) domains, cryptochrome 2 (CRY2) domains, and rhodopsin-type photoreceptors, which makes it a popular bioluminescence donor for coupling with optogenetic proteins. A mass of artificial CTZ analogs have been designed and synthesized in order to develop CTZ-type substrates with unique bioluminescence intensity and spectra, and a longer half-decay life [25,26,27,28,29,30,31,32]. Moreover, furimazine (FZ) is a potent substrate that has been adapted for NanoLuc involved in bioluminescence-optogenetics assays [18,19,21,22,23,33,34,35,36,37]. In addition to CTZ and FZ, coelenterazine h (CTZ h), diacetyl CTZ h, FZ derivative fluorofurimazine (FFz), and FZ-4377 have been reported to be applied in bioluminescence-induced optogenetic probes. However, neither other CTZ-type analogs nor natural derivatives such as Cypridina luciferin have been used as substrate in applications. Gaussia luciferase stems from the deep-sea copepod Gaussia princeps and was the first luciferase to be fused with photosensitive domains to bring an internal biological light source into optogenetic probes [17]. The Gaussia luciferase series can emit visible light when incubated with CTZ. Renilla luciferase series are usually applied in optogenetics. A variety of brighter mutants of the Renilla luciferase have been engineered over the last decade, which has enriched the bioluminescence toolbox. Compared to the Gaussia luciferases and Renilla luciferases, NanoLuc, a small engineered luciferase derived from a deep-sea shrimp, Oplophorus gracilirostris, generates much brighter and more sustained blue luminescence.

The D-luciferin and luciferase pair is another widely applicable bioluminescence system. In general, the luciferases from the firefly and from the click beetle take advantage of D-luciferin as a substrate to produce bioluminescence in the range of 530–650 in the presence of ATP, Mg^2+^, and oxygen. Many synthetic luciferin analogs and engineered luciferases with brighter and red-shifted emissions have sprung up over the past two decades [38]. D-luciferin analogs and D-luciferin-dependent luciferases are currently regarded as excellent tools for in vivo bioluminescence imaging, which provides powerful optical means for a better understanding of the relevant bioscience.

Unlike the coelenterazine–luciferase system and the D-luciferin–luciferase system, bacterial bioluminescence and fungal bioluminescence are self-sustained luminescence systems available in heterogeneous hosts. Thus far, only the bacterial luciferin and fungal luciferin biosynthesis pathways have been clearly revealed. The bacterial luciferase originates from luminous bacteria such as Photobacterium, Vibrio, and Xenorhabdus, and catalyzes the oxidation of a long-chain aldehyde and reduced riboflavin phosphate (FMNH_2_) to produce cyan light. The bacterial bioluminescence pathway is encoded in the lux operon that contains luciferase subunits, the fatty acid reductase complex, and a flavin reductase enzyme [4]. The fungal luciferin is identified as 3-hydroxyhispidin that is generated from caffeic acid catalyzed by the hispidin synthase (HispS) and hispidin-3-hydroxylase (H3H) in steps. Then, the luciferin is oxidized by luciferase to yield green light (520 nm) and caffeylpyruvate that can be converted to caffeic acid by caffeylpyruvate hydrolase (CPH) [39]. Both bacterial and fungal bioluminescence systems have been engineered to be used as imaging tools and have the potential to monitor intracellular processes and interactions.

Considering that the occurrence of bioluminescence is initialized from the internal luciferase–luciferin reaction and does not demand an external light source, this optical imaging has low background interference and be easily adapted for noninvasive monitoring of biological processes in small and large animals [40]. Luciferases have been used as reporters for biological functions for decades. Engineering efforts have presented a diverse group of luciferases with improved dynamic and spectral emission profiles. Additionally, luciferases have participated in a range of energy transfer reactions, such as the bioluminescence resonance energy transfer (BRET) system. Moreover, researchers have made efforts to develop luciferin analogs with better photophysical properties, especially for high-sensitivity in vivo bioluminescence imaging and single-cell imaging [1,3,25,26,27,30,31,32,38,41,42]. Bioluminescence probes based on caged luciferin play a role in illuminating the molecular mechanisms of biological processes, detecting metal ions in organisms, sensing biomarkers for dysfunction and disease, detecting bioactive small molecules, and screening drug candidates [10]. In brief, a bioluminescence toolbox that includes synthetic luciferins, engineered luciferases, probes, and sensors provides multicolor optical imaging for investigating biological processes at the molecular level and small animal models in real time. Recent advances in bioluminescence technology have integrated luciferases with a variety of light-sensitive proteins and molecules, not only for imaging and sensing but also for controlling biological processes [9]. The luciferase–luciferin pair, taking the place of external light sources, emits bioluminescence to trigger the photosensitive proteins to undergo conformational changes or cleavage reactions, which promote downstream gene transcription and signal transduction so as to potentially control intra- and intercellular activities.

### 1.2. Optogenetics and Photoswitchable Modules

Optogenetics utilizes genetically encoded light-activated proteins to spatiotemporally and precisely manipulate the activity of cells in living tissue and animal models with the use of light. Oesterhelt and Stoeckenius discovered the first microbial opsin, which is a rhodopsin-like protein from the purple membrane of Halobacterium halobium, in 1971 [43]. Then, a variety of photosensitive proteins were identified in the years that followed [44,45]. Photoswitchable probes have been developing rapidly in neuroscience, immunity, and other biological science fields over the past decade, especially since the algal protein channelrhodopsin-2 was first found to be expressed in mammalian neurons in response to light by Edward S. Boyden [46,47,48]. These tools show their power in regulating signaling networks with many advantages such as a superior temporal and spatial resolution, easy delivery, rapid reversibility, and fewer off-target side effects [49,50].

**Figure 1:**
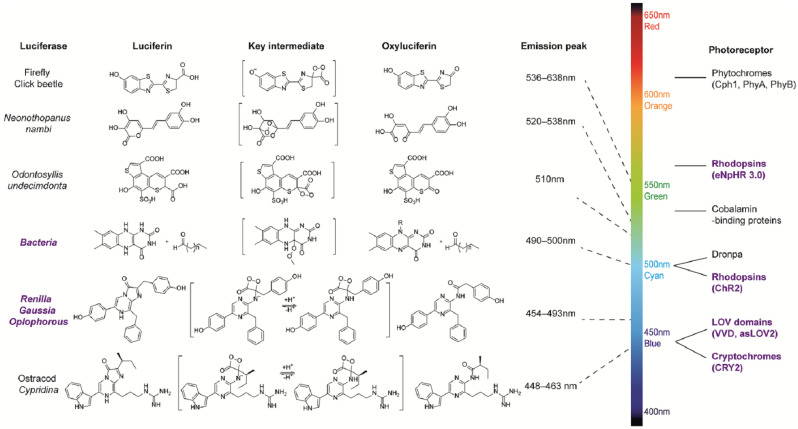
Bioluminescence systems and common photoreceptors.

Light-activated proteins with distinct structures and photon properties are key to the optical control of intracellular signal transduction. A variety of photoactivatable modules respond to light at varying wavelengths. These proteins change their conformation in response to light to trigger or block plentiful signaling pathways in vitro and in vivo. The known light-sensitive domains involved in optogenetics are LOV, CRY2, channelrhodopsin-2 (ChR2), and phytochrome B (PhyB) (Figure 1).

In brief, the mechanism behind photoswitchable probes involves a photoactivatable protein that is expressed in the target of interest and then responds to light to undergo conformational changes and emits light after absorbing energy from the excitation light, which leads to the turn on or turn off of ion channels, the modulation of inter- or intraprotein interactions, and downstream cellular responses. This permits observation or perturbation of selected cells in tissues and also connects the molecular mechanism with system-level behavior. There has been increasing success in using photoactivatable proteins to regulate signaling activities in living cells and free awake animals.

## 2. Bioluminescence-Induced Photoswitchable Protein-Based Optical Probes: A Novel Strategy for Sensing and Controlling Biological Processes

It is obvious that using bioluminescence to trigger optogenetic tools has advantages such as noninvasive detection and spatial and temporal precision. Berglund and co-workers fused channelrhodopsin-2 with Gaussia luciferase to create luminopsin (LMO) that responds to intrinsic biological light from the reaction of luciferase and coelenterazine [17]. Since then, the merging of bioluminescence and photosensory domains has become a novel strategy for the design and expansion of toolboxes for sensing and controlling biological processes, which is not only applied in neuroscience, but has also gradually come to play a role in other biological areas. These probes generally consist of two key components, the luciferase and the light-activated proteins in one molecule or in one system. Bioluminescence typically created by a coelenterazine-type luciferin–luciferase system triggers the photosensitive domains so as to drive downstream biological processes.

Luciferases emit bioluminescence in the presence of a chemical substrate, luciferin, which can be an alternative light source used to trigger light-sensitive domains. Additionally, bioluminescence, as a light source, has its own advantages such as avoiding tissue autofluorescence, excitation-induced tissue photodamage, and poor tissue penetration. According to previous studies, Renilla luciferase, Gaussia luciferase, and NanoLuc have often been chosen for use in combination with photosensitive proteins both with and without the use of fusion. Coelenterazine-type luciferin has often been used to trigger the process in a chemical way. The reason why they are popular could be that the bioluminescence spectra of Rluc, Gluc, and NanoLuc in the presence of CTZ analogs match the absorption spectra of photosensory domains such as ChR2 and LOV. These luciferases could perfectly serve as light donors that emit high bioluminescence, activating photoswitchable domains to modulate cellular activities and additionally enabling real-time imaging.

Below, we highlight the bioluminescence-driven optical probes (Table 1) for sensing biological interactions and signaling pathways, controlling cellular activities, and enabling synthetic biological activities, as well as their applications in physiology and pathology, and potential therapy.

### 2.1. Bioluminescence-Induced Optical Biosensors for Ion Sensing

Calcium ions (Ca^2+^), being universal intracellular secondary messengers, regulate abundant physiological processes. To achieve precise control over Ca^2+^ signaling, a variety of optogenetic probes have been crafted by coupling light-sensitive domains with intracellular signaling proteins to enable remote control of intracellular Ca^2+^ signaling both in cellulo and in vivo [68,69]. Bioluminescence-based Ca^2+^ imaging is a perfect strategy that is highly compatible with optogenetic actuators for imaging and monitoring of multiple cellular Ca^2+^ dynamics [70]. Undesirable consequences resulting from functional crosstalk between optogenetic light-sensitive domains can be avoided as bioluminescent indicators have self-luminescent excitation. The bioluminescent optogenetic Ca^2+^ sensors (Table 1) are helpful for the further study of Ca^2+^-related physiology, pathophysiology, and drug screening.

**Figure 2:**
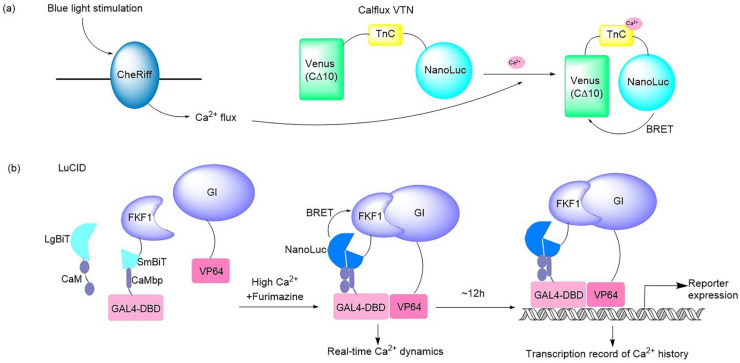
Illustrations of typical bioluminescent optogenetic Ca^2+^ sensors. (**a**) Schematic of BRET-based Ca^2+^ sensor Calflux VTN. Calflux VTN was designed by inserting troponin C domain between NanoLuc and Venus. The Ca^2+^ sensitive troponin undergoes a conformational change in response to binding Ca^2+^, which brings NanoLuc closer to Venus so that BRET can occur with a spectral shift [33]. (**b**) Schematic of dual Ca^2+^ indicator LuCID. In the presence of high Ca^2+^ and furimazine, BRET from NanoLuc activates the GI/FKF1 system, leading to the transcription of reporter genes. The real-time readout of Ca^2+^ activation is reflected via reconstitution of NanoBiT, while the steady recording of past Ca^2+^ activity is provided in the form of reporter expression controlled by the GI/FKF1 system [18].

It should be noted that a bioluminescent optogenetic opsin named bMCOII has been reported [21]. bMCOII was created by fusing the C-terminus of mutated opsins from algae (Chlamydomonas) with the N-terminus of a bioluminescent protein GeNL made of NanoLuc and GFP. The calmodulin-M13 domain fused with GeNL is located on the cytosolic side of the membrane and just beneath the bMCOII-TM actuator domain, which allows GeNL to bind to Ca^2+^ flowing into the cell when the actuator is evoked. Upon light stimulation, the transmembrane opsin channel is opened, allowing Ca^2+^ to enter the transfected cells, which can be detected and reported by the Ca^2+^-GeNL sensor immediately. This is a hybrid optogenetic actuator and a bioluminescence Ca^2+^ sensor, which enables simultaneous optical modulation and bioluminescence imaging of cortical activities in vitro and in vivo. Moreover, Johnson’s group developed a BRET-based Ca^2+^ sensor that can be partnered with optogenetic probes (Figure 2a). This BRET system consisted of bright NanoLuc luciferase in conjunction with the fluorescent protein Venus. The Ca^2+^ flux elicited by the optogenetic ChR2 domains and melanopsin in response to light in hippocampal and fibroblast neurons was imaged and quantified successfully by use of this Ca^2+^ sensor [33]. A dual-purpose probe named LuCID has recently been crafted to provide real-time dynamics and the transcriptional record of Ca^2+^ [18] (Figure 2b). The LuCID probe is constructed by inserting NanoBiT fusions into the GI/FKF1 system. In the presence of high Ca^2+^ and furimazine, BRET from NanoBiT activates the blue-light-induced protein dimerization system through the LOV domain in FKF1 [71], which leads to the transcription of reporter genes. The real-time readout of Ca^2+^ activation is reflected via reconstitution of NanoBiT, while the steady recording of past Ca^2+^ activity is provided in the form of reporter expression controlled by the GI/FKF1 system, enabling LuCID to function in both Ca^2+^ indicator and transcriptional Ca^2+^ integrator capacities in living cells. In addition to Ca^2+^ sensors, Inagaki and co-workers developed a bioluminescent NanoLuc protein-based voltage indicator, called LOTUS-V, which was combined with the optogenetic proteins ChR2 and eNpHR3.0 to allow long-term membrane voltage imaging in an in vitro cardiomyocyte model [34] and in vivo imaging of brain activity [35]. This is a useful live imaging tool with multipurpose applications, such as for studying cardiomyocyte behavior [34] and recording brain activity in multiple socially interactive animals [35].

The cooperation mode of the optogenetic component and luciferase sensor varies in different systems, and is mainly divided into two typical principles: in one case, the optical channel is triggered by light stimulation, resulting in Ca^2+^ current, which is detected and reported by the designed bioluminescent Ca^2+^-sensitive protein [21,33]. In the other case, the bioluminescence generated by the Ca^2+^ indicator binding with Ca^2+^ is responsible for stimulating optogenetic proteins, leading to a series of downstream reactions to record and display the dynamics of Ca^2+^ [18].

Regarding the experimental protocols for the application of typical bioluminescent optogenetic Ca^2+^ sensors, the bioluminescent optogenetic Ca^2+^ sensors may be a fusion-type protein constructed by optogenetic domains and bioluminescent Ca^2+^ indicators [18,21], or the two partners may be expressed separately in target cells by co-transfection [33,34,35]. The primary intended use of bioluminescent optogenetic sensors is for the imaging and recording of biological activities over a long period and in a noninvasive manner, as well as avoiding the undesirable effects of fluorescence irradiation such as tissue autofluorescence, excitation-induced tissue photodamage, and poor tissue penetration. The luciferase NanoLuc is commonly involved in bioluminescence Ca^2+^ sensors: its unique luciferin furimazine should be prepared for a substrate solution as a bioluminescence trigger [18,21,33,34,35]. Apart from this, other basic routes include cell preparation and transfection, virus preparation, virus injection to generate animal models, cranial surgery, brain slice preparation, imaging and recording by an EMCCD camera, and patch clamp/voltage clamp recording. Considering that Ca^2+^ signaling is not only important in neuronal functions but also plays a role in other biological processes such as cardiocyte activities, bioluminescent optogenetic Ca^2+^ sensors may allow a wide range of imaging and the quantification of live Ca^2+^ fluxes in multiple physiological and pathophysiological conditions.

In addition to the above, many bioluminescent indicators have been generated and have potential to be a partner of optogenetic probes. The Renilla luciferase and its improved variant Rluc8, NanoLuc, and the bioluminescent aequorin protein have been used in the construction of bioluminescent Ca^2+^ indicators. For example, a series of multicolor nanolanterns that resulted from the coupling of Rluc8 or NanoLuc luciferase with a fluorescent protein were demonstrated to image Ca^2+^ dynamics in the cytosol, nucleus, and mitochondria [72,73], as well as in the sarco/endoplasmic reticulum (SR/ER) [74]. Moreover, a high-affinity fluorescence/bioluminescence bimodal indicator that fused the Ca^2+^ sensing tool GCaMP6f to a NanoLuc-derived reporter NanoBiT was demonstrated to be useful for imaging cytosolic Ca^2+^ dynamics. Another low-affinity bimodal Ca^2+^ indicator variant that coupled the Ca^2+^ sensing tool R-CEPIA1er with NanoBiT was created to target Ca^2+^ imaging of ER [75]. A bioluminescent Ca^2+^ indicator, LUCI-GECO1, based on the coupling of BRET from the NanoLuc luciferase to a topological variant of GCaMP6s is reported to be able to image Ca^2+^ changes in cultured cells and primary neurons [76]. Tricoire and co-workers engineered a bioluminescent Ca^2+^ probe that consisted of a fusion of aequorin and GFP and could be used in whole-field bioluminescence imaging of neuronal network dynamics [77]. The function of bioluminescent indicators is not limited to ion sensing. For example, a stereospecific bioluminescent luciferase probe has been engineered to detect endogenous D-cysteine quantitatively in mammals. D-cysteine combines with 2-cyano-6-hydroxybenzothiazole (CHBT) after the addition of base and reducing agent to form D-luciferin, which can be used as the substrate of luciferase to make it emit light, allowing a noninvasive method to monitor D-cysteine and further demonstrate its function [78]. Based on BRET, Park and co-workers created an ERα dimerization assay and subsequent ERβ dimerization assay in human cells to elucidate the ER dimerization potential of estrogenic compounds [79,80]. Furthermore, NanoBiT subunits LgBiT and SmBiT were respectively fused to the human androgen receptor (hAR) to construct a system that images the activation state of ARs. When testosterone binds to the AR, a series of conformational changes cause dimerization of the hAR and the reconstruction of NanoBiT, which in turn will yield bioluminescence in the presence of the furimazine [81]. Accordingly, these bioluminescent indicators may represent maximal biocompatibility and the best signal-to-noise ratios, avoiding the interference from crosstalk between the optogenetic tools and fluorescent indicators when they are applied with optogenetic probes. However, a major limitation is that the consumption of the substrates could gradually reduce signal brightness [82], which results in partially sacrificing the spatial resolution [19,68].

Although optogenetic proteins have been widely used in eukaryotic systems, especially in neuroscience, a variety of photosensitive proteins have been discovered from microorganisms [46]. The renaissance of optogenetic applications in prokaryotes has been underway since synthetic sensors for light-induced gene expression were created with the use of photoreceptor Cph1 and the histidine kinase EnvZ in 2005 [83]. Optogenetic tools have been developed to manipulate molecular processes, interbacterial interactions, and cell-to-environment interactions in microbiology. A bacterial sensor for Hg^2+^ based on a lux bioluminescence-triggered photoswitchable magnet system was developed, which provides a new strategy for whole-cell biosensors [51]. When the Hg^2+^ binds to the transcriptional regulator MerR, the lux operon undergoes expression to emit bioluminescence, which leads to the activation of photoswitchable proteins that are displayed on bacterial surfaces. Thus, this bioluminescent optogenetic tool with the ability to control bacterial aggregation provides an alternative tool in the regulation of bacterial consortia.

### 2.2. Bioluminescence-Aided Optical Tools for Reprogramming Cellular Activities

According to the BRET mechanism, a luciferase could be engineered as a bioluminescent–fluorescent protein that emits light from cyan to red across the visible spectrum. Hence, the BRET strategy has the ability to alter the spectrum of a bioluminescent protein, which is beneficial to match more light-sensitive domains to the design of sophisticated bioluminescent optogenetic tools.

**Figure 3:**
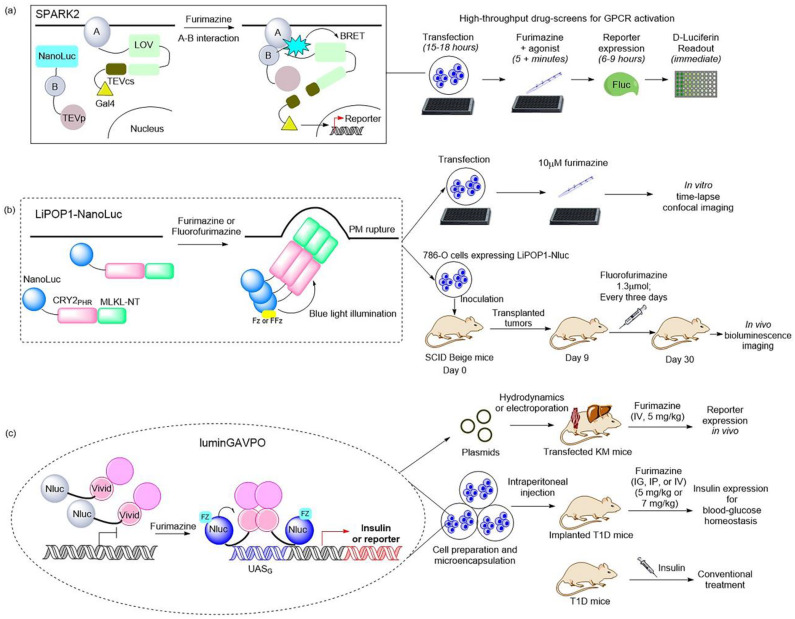
Illustrations of classic BRET-based photosensory probes. (**a**) Schematic of SPARK2 and its general flow for high-throughput GPCR agonist screening [19,84]. SPARK2 contains two modules: One is the fusion of target protein A, an LOV domain, and a transcription factor with a peptide linker including the TEVcs domain. The other is the fusion of target protein B, NanoLuc, and the TEV protease. When the target proteins are close enough to interact and the luciferin is provided, the BRET activates the LOV domain to expose TEVcs for protease cleavage in order to induce the release of transcription factors. (**b**) Schematic of LiPOP1 and its experimental procedure for controlling nonapoptotic cell death in vitro and in vivo [22]. LiPOP1–NanoLuc was generated by inserting NanoLuc into LiPOP1 between mCherry and CRY2. With the addition of the FFz, the blue bioluminescence catalyzed by NanoLuc stimulates CRY2 to undergo oligomerization, which causes MLKL to expose the N-terminal helix bundle domain and destroy the membrane. (**c**) Schematic of luminGAVPO and its application for pulsatile activation of transgene expression in mice [23]. luminGAVPO is the fusion of NanoLuc and a light-switchable transcription factor GAVPO. Light stemmed from the NanoLuc–furimazine reaction activates the VVD domain in the transactivator in order to cause dimerization and binding of luminGAVPO to the related promoter, thus leading to downstream gene transcription.

The BRET strategy has been applied in optogenetics to generate optogenetic probes for Ca^2+^ sensing [33] as mentioned above, and engineer inhibitory luminopsins [65] for controlling neuron activities (in Section 2.3). Recently, a series of novel and delicate BRET-based optogenetic tools have been reported (Table 1, Figure 3). NanoLuc is the preferred luciferase as a bioluminescence donor due to its ability to activate blue-to-green light-sensitive proteins via BRET, which results in downstream events. In 2018, Komatsu and co-workers developed a BRET–FRET hybrid biosensor named hyBRET that consists of luciferase Rluc8, a yellow fluorescent protein, and a cyan fluorescent protein for intramolecular ERK activity. This biosensor is compatible with the optogenetic protein CRY2 for imaging and monitoring ERK activation in live cells and in vivo. In this case, a prolonged half-life substrate diacetyl CTZ h was used to induce bioluminescence for longer in vivo imaging. It is a promising platform for the visualization of the multiscale dynamics of cell signaling, and even for the visualization of pharmacodynamics in living animals, which is helpful for drug development [52].

Moreover, a BRET-based optogenetic transcription reporter, named SPARK2, used for the quantitative detection of protein–protein interactions, has been reported [19,84] (Figure 3a). This transcription reporter has the ability to switch gates between external light and the addition of a luciferin for temporal specificity. NanoLuc was employed as a light-emitting moiety to control the blue-light-sensing LOV domain from Avena sativa (asLOV2), which yielded a lower background, leading to improved protein–protein interaction specificity. As regards the generality of SPARK2′s design, it contains two modules. One is the fusion of a target protein, an LOV domain, and a transcription factor with a peptide linker including the TEVcs domain for protease recognition. The other is the protein fused by a target protein, NanoLuc, and the TEVp protease. When the target proteins are close enough to interact and the luciferin is provided, the BRET activates the light-sensing LOV domain to expose the TEVcs site for protease cleavage in order to induce the release of transcription factors. Then, the downstream gene expression is regulated by the released transcription factors. This is the AND logic of SPARK2 for target protein–protein interaction. This tool has been applied in high-throughput screening for GPCR agonists and the study of transcellular interactions. For example, when SPARK2 was applied to detect the PPI interaction between the GPCR beta-2 adrenergic receptor (β2AR) and its cytosolic effector arrestin, the NanoLuc was fused to arrestin–TEVp and the LOV-sensing domain component was fused to β2AR; these two constructs were co-expressed with the gene reporter of citrine in HEK293T cells. With the addition of the β2AR agonist isoetharine and 10 μM furimazine for 15 min, robust citrine expression was observed. SPARK2 displayed furimazine- and agonist-dependent expression of citrine when it was designed for other GPCR/arrestin pairs, such as arginine vasopressin receptor 2 (AVPR2) and dopamine receptor type I (DRD1). Another similar BRET-activated optogenetic paradigm, BEACON, has been developed with the use of self-illuminating bioluminescent–fluorescent proteins (LumiFluor) [36] that were generated by fusing the N-terminus of NanoLuc to the C-terminus of fluorescent proteins eGFP or mCerulean3 with a flexible linker. The reaction of NanoLuc and furimazine resulted in biological light, and BRET was carried out to control a diverse variety of blue–green light-sensitive proteins, including CRY2, LOV, and VVD, which provided a novel strategy for achieving rapid and robust activation toward a variety of commonly used optogenetic systems in a spatiotemporally restricted manner. For example, the light emitted from LumiFluor upon the addition of 10 μM furimazine activated the cryptochrome-based CRY2–CIBN system, the LOV-based FKF1–GI and iLID system, and the VVD-based pMagnet system, then gene expression or protein co-localization was achieved. However, further studies are required to determine whether bioluminescence activates BEACON to trigger downstream processes in vivo.

Recently, NanoLuc and a novel furimazine analog, fluorofurimazine (FFz), were utilized to emit bioluminescence, which provided a biocompatible approach by which to effectively activate a novel optogenetic tool, LiPOP1, based on photosensitive domain CRY2 to achieve wireless control of tumor lysis in living animals [22] (Figure 3b). LiPOP1 is a fusion protein created by fusing the N-terminal of mCherry-CRY2 to the C-terminal of MLKL that plays a role in the execution of necroptosis. A hybrid fusion protein, LiPOP1–NanoLuc, was generated by inserting NanoLuc into LiPOP1 between mCherry and CRY2 in order to validate the bioluminescence-mediated optogenetic stimulation to activate LiPOP1 via CRY2 oligomerization. With the addition of the substrate FFz, the blue bioluminescence catalyzed by NanoLuc stimulates CRY2 to undergo monomer-to-oligomer transition, which also leads to the oligomerization of MLKL. Activated MLKL exposes the N-terminal helix bundle domain (HBD) and migrates to the plasma membrane, destroying the membrane and causing necroptosis. Additionally, LiPOP1–NanoLuc was demonstrated to be able to induce PM translocation and necroptosis in three cancer cells lines, including HeLa cells, B16 cells, and 786-O cells. Furthermore, human 786-O cells were engineered to express LiPOP1–NanoLuc, and these were subcutaneously inoculated into mice to generate a mouse xenograft model. The mice were treated with FFz (1.3 μmol/25 g) by intratumoral injection every three days for a total of seven injections, after which they displayed a significant reduction in tumor size. This tool will likely act as a synthetic light-switchable gene that allows selective killing of abnormal cells and the elimination of therapeutic cells after treatment in the future.

Furthermore, the description of NanoLuc-driven bioluminescence directly activating the light-switchable transcription factor that fused to NanoLuc was reported [23] (Figure 3c). This is a bioluminescence transcription factor, termed luminGAVPO, in which NanoLuc was fused to a light-switchable transcription factor GAVPO [85]. NanoLuc is close enough to VVD in this fusion protein to allow BRET to occur. Light stemmed from the NanoLuc–furimazine reaction and the external blue laser activated the light-sensitive VVD domain in the transactivator in order to cause dimerization of luminGAVPO and binding of luminGAVPO to the related promoter, thus leading to downstream gene transcription. When the bioluminescence was dimmed due to the consumption of luciferin, the luminGAVPO dimer gradually dissociated from the promoter, thereby stopping the transcription of the target gene. The downstream gene expression reached the highest level and was sustained for 2–3 h when the furimazine concentration was 2.5 μM in vitro. The pulse amplitude and duration of the transgene expression were consistent with the furimazine concentration, which is suitable for studying the pulsing dynamics of signaling proteins. Moreover, this tool was activated in a pulsatile manner by the administration of furimazine for target gene expression in transfected mice. As shown in a publication, the synthetic BRET-induced transgene expression system (LuminON) based on luminGAVPO for gene therapy was successfully used to mediate blood glucose homeostasis in a type 1 diabetic mouse model (T1D) in a pulsatile fashion. The stable cell line that expressed luminGAVPO-mediated insulin was engineered and microencapsulated into coherent, semipermeable, and immunoprotective alginate-poly-(L-lysine)-alginate beads. The T1D mice were intraperitoneally injected with microencapsulated transgenic cells and treated with 5 mg/kg furimazine, which resulted in significant restoration of blood glucose levels and enhanced glucose homeostasis. This study provided a promising BRET-based optogenetic tool for the precise control and a better understanding of the pulsing behaviors in pharmacological studies.

Another example is the light-inducible transcription factor EL222, which was directly controlled by its fused partner, Gaussia luciferase [53]. Unlike luminGAVPO used for gene therapy, this kind of bioluminescence- or BRET-induced transcription factor was successfully applied to achieve communication between synthetic cells and natural cells. Schroeder’s group engineered the high-level Gaussia luciferase-expressing synthetic cell to activate sensitive proteins such as retinal rhodopsins in order to induce photoconidation in the fungus Trichoderma atroviride. The bioluminescence generated in synthetic cells with the addition of 5 μM CTZ enabled intercellular signaling between a synthetic cell and a natural cell. Then, they utilized the light-dependent transcription mechanism mediated by the transcription factor EL222 and the BRET strategy to demonstrate intercellular signaling between light-producing synthetic cells and light-responsive natural cells. The synthetic cells were first engineered to contain the self-activating fusion proteins composed of Gluc on their N-terminal end connected through a flexible peptide linker to the light-inducible bacterial transcription factor EL222 on their C-terminal end. Native CTZ (0.2 nmol) was added every 30 min to the synthetic cell culture in a 384-well microplate to produce and maintain bioluminescence to activate the downstream transcription of the RFP protein. Then, they engineered another fusion protein: N-terminal Gluc fused to C-terminal iLID with a linker peptide and an N-terminal his-tag. The addition of CTZ led to a reaction with the Gluc being localized in the synthetic cells’ membranes to emit bioluminescence, which stimulated iLID and then activated membrane recruitment of sspB-tagged proteins to Gluc-iLID-labeled synthetic cells. Additionally, these BRET-based optogenetic fusion proteins could be imaged simultaneously during the activation process with the use of a microscope, providing spatial information about the activation. Their study to facilitate communication between synthetic cells and natural cells holds promise for deploying synthetic cells as a tunable and embeddable tool by which to control engineered processes inside tissues.

Additionally, a bioluminescence-activated optogenetic tool, bPAC–nLuc for cAMP synthesis, was generated by fusing a light-activated adenylyl cyclase from Beggiatoa with a myc tag to NanoLuc [37]. This tool can be precisely activated by furimazine and blue light to regulate cAMP production temporally and spatially in cellulo, which means that it is adaptable in imitating physiological levels and maintains cAMP synthesis to control downstream processes in living cells. For instance, the PCCL3 rat thyroid cell stably expressing bPAC–nLuc was engineered to verify this probe’s function and downstream signaling. Upon the addition of Fz or its analog Fz-4377 with long-lasting luminescence and lower toxicity, bPAC–nLuc is activated and sustained to continuously synthesize cAMP localized in the cytosol or nucleus, which leads to cAMP-dependent thyroid cell proliferation. Considering that aberrant cAMP signaling is linked to a variety of diseases such as cancer and heart disease, this tool may play a role in the study of cAMP-involved biology and pharmacological interventions.

Although the cellular activities regulated by these systems are different, their control principles can all be attributed to the optogenetic programming of living organisms using genetically encoded bioluminescence systems. When the substrate is added, the bioluminescence catalyzed by luciferase triggers the light-switchable module to undergo conformational change or expose the cleavage site, leading to the reprogramming of downstream signal transduction and gene expression.

The general procedure for the application of the tools in this section essentially includes cell culture and transfection, luciferin solution preparation, estimation of energy transfer efficiencies, compatibility tests with optogenetic tools, bioluminescence activation and imaging in vitro, tests for specific signaling molecules, animal preparation, and bioluminescence activation and imaging in vivo. In general, researchers have designed these tools as fusion proteins consisting of luciferase and light-switchable domains. Most of these BRET-optogenetic probes and platforms discussed in this section are not designed for neuronal control, but are fit for the study of protein–protein interaction and drug screening (e.g., hyBRET, SPARK2, and BEACON), gene therapy (e.g., LiPOP–NanoLuc and luminGAVPO), and synthetic biological activities (reference [53] and bPAC–nLuc). Their application could be carried out using plate readers and an imaging system with a CCD camera. When they are applied in animals, surgery protocols such as craniotomy are not needed, but transgenic cell line-derived xenograft models are often generated. Considering that most of them can be expressed in many cell lines using common transfection methods, and that their “on–off” switch can be induced by chemical substrates more conveniently, they may be adapted for the high-throughput screening method of target molecules and can expand the boundaries of gene therapy in future applications.

### 2.3. Luminopsins: Bioluminescent Optogenetics Probes in Neuroscience 

**Figure 4:**
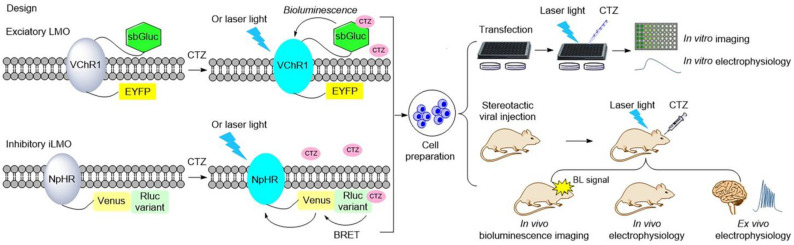
Illustration of typical luminopsins [17,54,55,56,58,59,65,87] and the general experimental procedure for studying and controlling neural activity in vitro and in vivo. Luminopsins are triggered by bioluminescence or bioluminescence resonance energy transfer (BRET) with the addition of luciferin. They can also be activated by laser light.

Luminopsins are a fusion of light-emitting luciferases and light-sensing opsins, which results in optical and chemical control in one molecule. The advantages of utilizing luminopsins are obvious: they allow tracking of the signaling with spatial and temporal precision in a noninvasive manner, which bypasses the major challenge of implanting an external light source. To date, luminopsins have been successfully applied in the modulation of neuronal activity and intracellular signaling at different temporal and spatial resolutions. Some typical luminopsins and their applications are shown as follows (Table 1, Figure 4).

In 2013, the Hochgeschwender group first reported the development of the fusion of Gaussia luciferase and channelrhodopsin as a luminopsin for use in the manipulation of neuronal activity. The light-activated ion channel, channelrhodopsin-2 from Chlamydomonas (ChR2), and Gaussia luciferase (Gluc), a secreted form of luciferase from the marine copepod Gaussia princeps, were chosen to be fused for the creation of the luminopsin LMO. The addition of coelenterazine (CTZ), the substrate of luciferase Gluc, results in sufficient bioluminescence to activate the coupled ChR2, which in turn leads to the influx of a large number of extracellular cations to produce action potential [17,86]. Another more efficient bioluminescence-aided optogenetic probe, LMO2, that consists of the red-shifted and more sensitive Volvox channelrhodopsin-1 (VChR1) and Gaussia luciferase, was further developed [17]. The latter was able to modulate the intrinsic excitability of neurons. Then, this bioluminescent-driven optogenetic tool, LMO2, was applied in noninvasive in vivo imaging and modulation of neuronal activity in mice [54]. When the luciferin CTZ was intravenously administrated and reached the brain, it reacted with Gluc to produce bioluminescence, which allowed imaging of the neurons and illuminated channelrhodopsin to alter neuronal activity.

Additionally, the Gross group reported an inhibitory luminopsin (iLMO) series that consisted of Renilla luciferase (Rluc) and Natronomonas halorhodopsin (NpHR) and its means of suppressing neural activity in vitro and in vivo [65]. The luciferases were coupled to light-sensitive opsin NpHR to generate iLMO1 and iLMO2, respectively. Their study showed that iLMO2 had the robust effect of suppressing action potential firing and synchronous bursting in the entire neural network in vitro and in vivo. This inhibitory luminopsin was found to have the ability to modulate neural activity in freely behaving animals and target specific sites in the brain in a barely invasive manner. Further, iLMO2 was utilized in the optogenetic modulation of multiple nodes in an epileptic network for noninvasive seizure suppression, which provided a unique approach for demonstrating the interrogation mechanism of neural networks and treating neurological diseases involving broad neural circuits [66]. Moreover, iLMO2 was applied in the inhibition of motor neuron activity in a noninvasive way, which played a role in addressing the mechanism of motor axon regeneration by exercise and recovery of function after peripheral nerve injury [67].

In 2016, Berglund et al. promoted the development of luminopsins by fusing the bright Gaussia luciferase variant (sbGluc) with Volvox channelrhodopsin-1 (VChR1) to create the luminopsin LMO3 with improved light emission, which resulted in a higher photocurrent for the modulation of neuronal activity in vitro and in vivo [55]. Another inhibitory luminopsin named iLMO was formed by use of photosensitive proton pumps from the fungus Leptosphaeria maculans (Mac) and the Gaussia luciferase variant slGluc, described in the same publication. Their study indicated that both of them were able to act as promising probes for the control of neuronal behavior in vitro and in vivo [55].

A few years later, the luminopsin LMO3 was applied in cell transplantation therapy to enhance neuronal repair, neural network connections, and functional recovery after ischemic stroke in a mouse model [56]. Thus, these transplanted cells containing a bioluminescent optogenetic tool provide a novel and promising treatment for neuronal functional recovery. Subsequently, Zenchak et al. demonstrated that the activation of LMO3 by CTZ led to stimulation of transplanted neural precursor cells, which improved motor skills in a mouse model of Parkinson’s disease [57]. Moreover, LMO3 and iLMO were employed as a pair of probes to manipulate neuronal activity in hippocampal CA3 and were found to remarkably affect spatial working memory and spatial and episodic short-term memory, but rarely affected either spatial or episodic long-term memory [58]. As the dysfunction of working memory and short-term memory is correlated to mild Alzheimer’s disease (AD), this application of excitatory and inhibitory luminopsins in studying the function of CA3 in working memory may be helpful for treatment in the early stages of AD. Furthermore, LMO3 was utilized as a tool to rehabilitate spinal cord injury in a mouse model, which provides a foundation for the use of bioluminescent optogenetic treatment for functional recovery after severe spinal cord injury [59]. Additionally, LMO3 was also employed in noninvasive manipulation and imaging of neurons for postnatal brain development in mice [87].

Since LMO3 is a promising tool involved in neurological disorders, scientists are focusing on investigating the optogenetic mechanism driven by bioluminescent photon emission. Gomez-Ramirez et al. characterized the relationship between bioluminescent optogenetic effects and the bioluminescent photon emission in the neocortex in vivo [88]. Further, Zhang et al. engineered an enhanced iteration of LMO3, named eLMO3, with improved membrane trafficking, which resulted in a significant increase in photocurrents and more efficient control of neuronal activity for functional interrogation such as the examination of whisker-response effects [60]. Subsequently, the bioluminescent optogenetics tool eLMO3 has been used to enhance axon regeneration after peripheral nerve injury [61,62]. Recently, LMO3.2 was designed by replacing the Volvox channel used in LMO3 with a more sensitive blue-shifted opsin from Scherffelia, which resulted in a four-fold higher response after being induced by CTZ. With the increased light sensitivity and accelerated response time, LMO3.2 has the capacity to efficiently control neuronal activity by noninvasive modulation and improve locomotor function after thoracic spinal cord injury [63]. Moreover, luminopsin 4 (LMO4) was created by fusing the brighter Gaussia luciferase mutant GlucM23 with the optogenetic element Volvox channelrhodopsin-1 (VChR1). Additionally, the combination of GlucM23 with anion channelrhodopsin iChloC produced inhibitory luminopsin 4 (iLMO4). The authors demonstrated that both LMO4 and iLMO4 were efficient probes for bimodal opto- and chemogenetic control of neural activity in a mouse model [20]. In 2020, Berglund et al. incorporated channelrhodopsin-2 with step-function mutations as the light-sensing opsin moiety and sbGluc as the light-emitting luciferase in a new luminopsin fusion protein, named step-function luminopsin (SFLMO), which has diversified the toolbox of bioluminescence optogenetic probes for bimodal control of neuromodulation for functional interrogation or therapeutic purposes [64]. Furthermore, scientists have also employed both the optogenetics approach and bioluminescence reporters to investigate mammalian circadian rhythms [89,90,91].

Currently, the luminopsin toolbox contains excitatory, inhibitory, and step-function luminopsins. They are triggered both optically by light and chemically by administration of substrates and have been successfully applied in the bimodal modulation of neural activities in vitro and in vivo, especially in the interrogation of neurons in freely moving animals. Several studies of the past few years have indicated that luminopsins and the derived platforms allow noninvasive and remote control of transplanted stem cells’ activities in neurological disorders, which can potentially provide an important approach to the therapy of neurodegenerative diseases, including Parkinson’s disease, Huntington’s disease, and Alzheimer’s disease.

Apart from luminopsins, Hochgeschwender and co-workers designed an ingenious bioluminescence-driven optogenetic system that couples luciferase with photoreceptors without fusion. They took advantage of light-emitting luciferase sbGluc that is released from presynaptic vesicles and produces interluminescence to activate postsynaptic excitatory or inhibitory opsin, thus providing a platform by which to interrogate specific neural circuits with temporal and spatial control in vivo [92].

Almost all luminopsins have been developed for the study and modulation of neuronal activity in the intact living brains of freely behaving animals. Luminopsins and luminopsin-type proteins share similar molecular designs and general experimental procedures for their application in cells and organisms.

All luminopsins and inhibitory luminopsins are the fusion proteins of blue light-emitting luciferases or luciferase-fluorescent proteins paired with the blue light-sensing photoreceptors. Specifically, LMO1 (Gluc–ChR2–EYFP), LMO2 (Gluc–VChR1–EYFP), LMO3 (sbGluc–VChR1–EYFP), and LMO4 (GlucM23–VChR1–EYFP) share the same fusion design as that of the Gluc-type luciferase, which is fused to the N-terminus of the ChR domain, while the fluorescent protein EYFP is fused to the C-terminus of the ChR domain. eLMO3 was created by inserting the Golgi trafficking signal from a neuronal potassium channel into the position between VChR1 and EYFP in the LMO3 fusion protein. The step-function series luminopsin fusion proteins were generated by coupling the luciferase mutant sbGluc to the N-terminal of the ChR2 mutation. With regard to the design and construction of inhibitory luminopsins, TagRFP–Rluc is coupled to the C-terminus of NpHR to create iLMO1. The TagRFP–Rluc in iLMO1 is replaced by a nanolantern to create iLMO2. The design of iLMO4 (GlucM23–iChloC–EYFP) is similar to that of LMO4; the GlucM23 luciferase mutant is fused to the N-terminus of iChloC, and the fluorescent protein EYFP is fused to the C-terminus of iChloC. Likewise, iLMO is a fusion protein of slGluc and Mac-EYFP.

Regarding the general approaches of these tools, the basic procedures include cell culture and transfection, luciferin solution preparation, bioluminescence imaging and activation in vitro, animal preparation, bioluminescence activation in vivo, and surgery protocols. When these luminopsin-type fusion proteins are expressed in neurons, bioluminescence from the reaction of luciferase and luciferin can excite or silence neuronal activity in cultured neurons and in brain slices, and can elicit certain behaviors in freely moving mice. In addition to general approaches for the application of luminopsins, the detailed experimental protocol and tips for LMO3 application in the study of developing neural circuits in postnatal mice can be found in Crsepo’s protocol [87]. Tips regarding methodological procedures for LMO and iLMO (iLMO2, LMO3, or eLMO3) when addressing neuronal repair and functional regeneration after peripheral nerve injury [61,67], spinal cord injury [59], and ischemic stroke [56] can be found in related articles. Furthermore, tips for implementing the light-sensitive opsins ChR2 and NpHR in studies of circadian behavior and physiology including surgical procedures can be found in Jones’s protocol [89].

## 3. How to Optimize the Bioluminescence-Aided Photosensitive Probes

The key components of bioluminescence-driven optogenetic systems are luciferase and light-sensitive opsin. Their appropriate coupling, with or without fusion, will generate effective probes. There are several strategies that can be used to design and optimize them. The first is to select luciferase/luciferin with bright emissions and matchable bioluminescent spectra. At present, the luciferases and luciferins applied in optogenetics are basically derived from the coelenterazine–luciferase system (Table 2). For instance, the Gaussia luciferase/CTZ pair emits visible and robust bioluminescence with a peak emission of 480 nm, which is suitable to induce the channelrhodopsin-type opsin. When the luciferases are modified, mutants are likely to generate much stronger light. Some mutations of Gaussia luciferase and Renilla luciferase have been reported with excellent properties as compared to those of native types [3,30,32,70,93]. Additionally, the engineered luciferase NanoLuc and its unique substrate furimazine are another pair that match the absorption of photoreceptors such as LOV2 and ChR2. Furthermore, the optimization of CTZ analogs is helpful for achieving more efficient substrates so as to produce brighter emissions with luciferase, which may result in more efficient photoswitching of the optogenetic elements. CTZ-type luciferins, which are popular chemical substrates, have the ability of crossing the blood–brain barrier into brain to trigger the luminopsins to control neuronal modulation in freely moving animals [54,55,57,61,62,63,66,67]. Only CTZ, CTZ h, furimazine, and its analog FFz are applied in bioluminescence-aided optogenetics, even though many CTZ analogs have been developed. Hence, this should draw chemists’ attention to designing and grasping CTZ-type analogs with increased luminescent output and high stability for bioluminescence and increased water solubility [94].

Although the emission of bacterial bioluminescence with a peak of 490 nm has potential to trigger photosensitive LOV, ChR2, and CRY2 domains, bacterial bioluminescence has been rarely utilized in optogenetic probes. Bacterial luciferin biosynthesis is clearly known, and the pathway is encoded by the lux operon, which results in self-sustained light emission [4]. Additionally, the fungal bioluminescence pathway has been recently elucidated and emits autonomous green light with a peak of 520 nm [39], which may match some photoreceptors. Hence, it is well worth developing self-sustained bioluminescence systems to generate a stable cyan light source to control the optogenetic platform. There is another efficient luciferase, namely, firefly luciferase, that catalyzes D-luciferin to emit strong and red-shifted bioluminescence in the presence of ATP, Mg^2+^, and oxygen. A series of Fluc mutants with red-shifted and stronger emission have emerged [32,93]. However, firefly bioluminescence has not been utilized to trigger optogenetic domains. If the red-shifted luciferase is coupled to suitable light-sensitive opsins to create novel bimodal optical probes that are triggered by red light, it would improve and extend their application in vivo. In other words, more efforts need to be made to discover the means of engineering luminopsin that can be activated by red and near–far-red bioluminescence.

Many BRET-based optogenetic tools have been reported and are discussed in Section 2. A luciferase or a fusion of luciferase and a fluorescent protein, acting as a donor, can be fused with the protein of interest, protein a. Additionally, an optogenetic protein as a photoreceptor can be fused with the protein of interest, protein b, that has the ability to interact with the protein of interest, protein a. This is a useful way to detect protein–protein interactions and to sense intracellular and intercellular signaling pathways. The BRET strategy between a luciferase and a fluorescent protein allows flexibility of emission wavelengths that match the absorption of optogenetic proteins, which may inspire biologists to focus their attention on extending BRET-based optogenetic tools.

Furthermore, modification of light-activated opsins is another way to improve the efficiency of bioluminescence-aided optogenetic probes. The ideal photoactivatable protein should have the ability to remain open for longer periods after activation, being more sensitive to the response to light and producing larger photocurrents [95,96]. Moreover, variants with red-shifted absorption are more suitable for imaging and controlling through deep tissue. For instance, a great deal of application-specific ChR mutants with an accelerated response time, fast photocurrents, and a broader spectral range have emerged [97,98] which provide more choices for combination with luciferases.

It is also worth noting that with the rapid development of nanotechnology, an increasing number of biocompatible nanomaterials have played essential roles in sensing and controlling cell activities, combined with optogenetic tools. For example, UCNPs are able to convert NIR light into ultraviolet or visible light in the range of 300–800 nm that can activate most existing optogenetic constructs [99]. UCNP-based nanooptogenetics has been applied in optogenetic immunotherapies and modulation of LiPOP-mediated nonapoptotic cell death [100,101,102]. Photothermal nanoparticles such as AuNPs could convert light into thermal energy, which allow them to not only act as a carrier for plasmid delivery, but also convert NIR light into localized heat to induce Cas9 expression, subsequently driving the process of CRISPR genome editing [103]. In addition, other nanomaterials such as mechanoluminescent nanoparticles and radioluminescent nanoparticles have been applied in modulating neural activity with the cooperation of optogenetic proteins [104,105]. Compared to luciferase–luciferin systems, nanoparticles attach to the cell surface stably and do not require substrate injection, which means they could ensure higher spatiotemporal precision. Perhaps the intervention of nanoparticles has the potential to accelerate the optimization of bioluminescence-driven optogenetic systems and broaden their application.

**Table 2:** The comparison of classic blue-light-emitting luciferases.

Blue-Light-Emitting Luciferases	Peak Wavelength	Relative Brightness *	Decay Time	Potential Photoreceptor	In Application with Photosensory Domains	Reference
Rluc–CTZ	482 nm	1	0.6 h	Dronpa, rhodopsins, LOV domains, cryptochromes	iLMO1, hyBRET	[106,107]
Rluc8–CTZ	487 nm	4	>86 h		hyBRET	[107,108]
Gluc–CTZ	480 nm	24	1.3 ± 0.3 min		LMO1, LMO2	[20,109]
sbGluc–CTZ	480 nm	216	14.1 ± 3.2 min		LMO3, LMO3.2, eLMO3, SFLMO series	[20,109]
GlucM23–CTZ	480 nm	240	Not clear		LMO4, iLMO4	[20,110]
slGluc–CTZ	481 nm	72	Not clear		iLMO	[20,111]
LuxCDABE– Decanal and FMN	490 nm	/	/		Bacterial biosensors for mercury	[112]
NanoLuc–Fz	456 nm	96	2–3 h	LOV domains, cryptochromes, rhodopsins	bMCOⅡ, SPAPK2, LOTUS-V, BEACON, LiPOP, luminGAVPO, bPAC-nLuc	[113]
TeLuc–DTZ	502 nm	240	~40 min	Dronpa, rhodopsins	Not clear	[32]
RLuc8.6–CTZ	535 nm	6	Not clear	Rhodopsins, cobalamin-binding protein	Not clear	[108]

* Intensity values normalized to Rluc–CTZ under comparable conditions.

## 4. Conclusions and Future Directions

Here, we give an overview of coelenterazine-type bioluminescence-induced photosensory protein-based optical biosensors, highlighting their versatility and advantages, from their ability for sensing and controlling signaling pathways to their potential for reprogramming cellular behaviors. We have discussed the delicate design of these tools, their growing applications in investigating physiology and pathology, and their ability to shed light on potential therapeutic agents.

A bioluminescence-induced photosensory protein-based probe, as a new powerful strategy in life biosciences, has been successfully used to reveal and control the behaviors of ion channels, signaling pathways, and cellular activities, especially in vivo as mentioned earlier. It has considerable application potential in ion sensing, especially Ca^2+^ sensing via fusion or co-transfection of bioluminescent Ca^2+^ indicators and optogenetic domains, offering new possibilities among the constellation of Ca^2+^ detection methods and providing a creative idea to measure and study ion signals in living cells. With the advantage of high temporal and spatial resolution and superior flexibility in in vivo experiments, bioluminescence-aided optical probes also play an important role in reprogramming cell activities and molecular interactions. The coupling of coelenterazine-type biolminescence and photoswitchable domains provides the ability to genetically control protein–protein interactions, intracellular pathways, and even cell-to-cell communications. The in-depth findings of pathological processes and cellular interactions will be of benefit to the design of drug screening methods and even in the reprogramming of artificial cellular behaviors as potential therapy in the future.

Moreover, bioluminescent optogenetics probes have exerted a significant impact on neuronal science by allowing the visualization and manipulation of neuronal firing at the cellular, circuit, and system levels, which makes them a new and promising tool in the study of molecular mechanisms of neurological disorders. In particular, luminopsins have their own advantages in that they are encoded and fused with light-sensitive opsin so as to make use of the internal light that is generated by bioluminescent reactions, thus bypassing the major hurdle of traditional optogenetic probes to deliver external light into the brain. There is no doubt that understanding the molecular mechanisms of brain diseases is helpful for target identification and validation, as well as drug discovery and therapy development.

Although coelenterazine-type bioluminescence-aided photosensory protein-based optical biosensors have the advantage of broadening photosensory probes to nearly all live cells, tissues, and organs where the substrate spreads, they likely sacrifice spatial resolution because signals dim as a result of the gradual consumption of the substrate. Future efforts from crossdisciplinary researchers in biology, chemistry, and engineering may offer solutions to this problem. Additionally, the development of bioluminescence-driven optical biosensors will benefit from the optimization of luciferase–luciferin pairs, the modification of photosensitive proteins and their conjunction with luciferases, and their association with other strategies such as BRET. Ultimately, innovative and creative bioluminescence-induced optical tools have been successfully applied in sensing and controlling signaling pathways and cellular activity, which has provided molecular mechanisms and design strategies for future applications in target identification, drug screening, and gene therapy.

## Figures and Tables

**Table 1:** The classic bioluminescence-induced optical probes mentioned in this review.

Name	Coupling Method	Photoreceptor	Luciferase	Luciferin	Intracellular Effect	Purpose	Reference
bMCOII	Fusion	Mutant opsin from algae	GFP–NanoLuc	FZ	Ca^2+^ sensing	Cortical activities	[21]
/	Co-transfection	ChR2, melanopsin	Venus–NanoLuc	FZ	Ca^2+^ sensing	Ca^2+^ signaling in neuron	[33]
LuCID	Fusion	LOV domain in FKF1	Reconstituted NanoLuc (NanoBiT)	FZ	Ca^2+^ sensing	Study calcium signaling	[18]
LOTUS-V	Co-transfection	ChR2, NpHR3.0	NanoLuc–Venus	FZ	Voltage indicator	Imaging	[34,35]
/	Nonfusion	Magnets (from VVD)	LuxCDABE	Decanal and FMN	Bacterial adhesions	Hg^2+^ sensing	[51]
hyBRET	Co-transfection	CRY2	YFP–CFP–Rluc8	CTZh, diacetyl CTZ h	ERK activation	ERK activity	[52]
SPARK2	Co-transfection	asLOV2 (LOV from Avena sativa)	NanoLuc	FZ	Protein dissociation/transcription	Screening for GPCR agonists	[19]
BEACON	Co-transfection	CRY2, LOV, VVD	mCerulean3–NanoLuc, eGFP–NanoLuc	FZ	Transgene expression	Study signaling pathway	[36]
LiPOP	Fusion	CRY2	NanoLuc	FZ, FFz	Cell death	Nonapoptotic cell death	[22]
luminGAVPO	Fusion	VVD domain in transcription factor	NanoLuc	FZ	Transgene expression	Type 1 diabetes	[23]
/	Fusion	LOV domain in EL222	Gluc	CTZ	Cell communications	Control synthetic processes	[53]
bPAC–nLuc	Fusion	A light-activated adenylyl cyclase from Beggiatoa	NanoLuc	FZ, Fz-4377, CTZ h	cAMP synthesis	Control cAMP signaling	[37]
LMO1	Fusion	ChR2	Gluc	CTZ	Excite neurons	Control neuronal activity	[17,54]
LMO2	Fusion	VChR1	Gluc	CTZ	Excite neurons		
LMO3	Fusion	VChR1	sbGluc	CTZ	Excite neurons	Treatment for Alzheimer’s disease, Parkinson’s disease, and spinal cord injury. Study for postnatal brain development	[55,56,57,58,59]
eLMO3	Fusion	VChR1	sbGluc	CTZ	Excite neurons	Control neuronal activity, enhance axon regeneration after peripheral nerve injury	[60,61,62]
LMO3.2	Fusion	Opsin CheRiff	sbGluc	CTZ	Excite neurons	Control neuronal activity. Treatment for spinal cord injury	[63]
LMO4	Fusion	VChR1	GlucM23	CTZ	Excite neurons	Control neuronal activity	[20]
SFLMO series	Fusion	ChR2CS, ChR2DA, ChR2CS/DA	sbGluc	CTZ	Excite neurons	Epileptic networks	[64]
iLMO	Fusion	Mac	slGluc	CTZ	Inhibit neurons	Treatment for Alzheimer’s disease	[55,58]
iLMO1	Fusion	NpHR	TagRFP–Rluc	CTZ	Inhibit neurons	Control neuronal activity	[65]
iLMO2	Fusion	NpHR	Nanolantern (Venus–Rluc8)	CTZ	Inhibit neurons	Control neuronal activity, functional recovery after peripheral nerve injury	[65,66,67]
iLMO4	Fusion	iChloC	GLucM23	CTZ	Inhibit neurons	Control neuronal activity	[20]

## Abbreviations

AVPR2	arginine vasopressin receptor 2
AD	Alzheimer’s disease
AuNP	gold nanoparticle
asLOV2	LOV domain from Avena sativa
bMCOII	bioluminescence multicharacteristic opsin II
BRET	bioluminescence resonance energy transfer
BEACON	BRET-activated optogenetics
bPAC	photoactivated adenylyl cyclase from Beggiatoa
CTZ	coelenterazine
CRY2	cryptochrome 2
ChR2	channelrhodopsin-2
CPH	caffeylpyruvate hydrolase
Cph1	cyanobacterial phytochrome 1
CHBT	2-cyano-6-hydroxybenzothiazole
CalfluxVTN	calcium flux composed of Venus, troponin, and NanoLuc
CaM	calmodulin
CaMbp	calmodulin binding peptide
cAMP	cyclic AMP
DRD1	dopamine receptor type I
DBD	DNA binding domain
ERα/β	estrogen receptor alpha/beta
ER	endoplasmic reticulum
ERK	extracellular signal-regulated kinase
EYFP	enhanced yellow fluorescent protein
EMCCD	electron-multiplying CCD
eNpHR	enhanced Natronomonas halorhodopsin
eLMO	excitatory luminopsin
FZ	furimazine
FFz	fluorofurimazine
FLuc	firefly luciferase
GFP	green fluorescent protein
GPCR	G protein-coupled receptor
GLuc	Gaussia luciferase
GeNL	green nanolantern
HispS	hispidin synthase
H3H	hispidin-3-hydroxylase
HBD	helix bundle domain
hAR	human androgen receptor
iLID	improved light-induced dimer
iLMO	inhibitory LMO
LOV	light-oxygen-voltage
LMO	luminopsin
LuCID	luciferin- and calcium-induced dimerization
LiPOP1	light-induced nonapoptotic tool 1
luminGAVPO	luminescent transcription factor GAVPO
MLKL	mixed-lineage kinase domain-like pseudokinase
Mac	Leptosphaeria maculans
NanoBiT	NanoLuc binary technology
NpHR	Natronomonas halorhodopsin
nLuc	nanoluciferase
Phy	phytochrome
PHR	photolyase-homologous region
Rluc	Renilla luciferase
RFP	red fluorescent protein
SR/ER	sarco/endoplasmic reticulum
SPARK2	Specific protein association tool giving transcriptional readout with rapid kinetics
SFLMO	step-function luminopsin
sbGLuc	slow burn Gluc
slGLuc	super luminescent Gluc
TEVcs	tobacco etch virus cleavage site
T1D	type 1 diabetes
TnC	troponin C domain
UCNPs	upconversion nanoparticles
VVD	photoreceptor vivid
VChR1	Volvox channelrhodopsin-1
